# Obesity and Appetite Control

**DOI:** 10.1155/2012/824305

**Published:** 2012-08-01

**Authors:** Keisuke Suzuki, Channa N. Jayasena, Stephen R. Bloom

**Affiliations:** Section of Investigative Medicine, Imperial College London, Commonwealth Building, Du Cane Road, London W12 0NN, UK

## Abstract

Obesity is one of the major challenges to human health worldwide; however, there are currently no effective pharmacological interventions for obesity. Recent studies have improved our understanding of energy homeostasis by identifying sophisticated neurohumoral networks which convey signals between the brain and gut in order to control food intake. The hypothalamus is a key region which possesses reciprocal connections between the higher cortical centres such as reward-related limbic pathways, and the brainstem. Furthermore, the hypothalamus integrates a number of peripheral signals which modulate food intake and energy expenditure. Gut hormones, such as peptide YY, pancreatic polypeptide, glucagon-like peptide-1, oxyntomodulin, and ghrelin, are modulated by acute food ingestion. In contrast, adiposity signals such as leptin and insulin are implicated in both short- and long-term energy homeostasis. In this paper, we focus on the role of gut hormones and their related neuronal networks (the gut-brain axis) in appetite control, and their potentials as novel therapies for obesity.

## 1. Introduction

Despite recent progress in our understanding of the physiological mechanisms regulating body weight and energy expenditure, obesity remains a major worldwide health crisis with an array of vascular, metabolic, and psychosocial consequences [1, 2]. Overweight or obese individuals (body mass index 25–30) have an increased risk of developing diabetes, coronary heart disease, and hypertension [2, 3]. Adults with a body mass index of 40 or higher have been associated with a high risk of developing diabetes, hypertension, dyslipidaemia, asthma, arthritis, and poor health status, when compared with normal weight individuals [4].

Body weight is tightly regulated by complex homeostatic mechanisms. Obesity is a state in which energy intake chronically exceeds energy expenditure. Even a subtle mismatch (less than 0.5%) in caloric intake over expenditure is sufficient to cause weight gain [5]. The rising prevalence of obesity is likely to result from contemporary environmental and lifestyle factors such as increased access to palatable foods and reduced requirements for physical exercise, when compared with ancient hunter-gatherer lifestyles characterised by unpredictable periods of feast and famine.

In addition to local paracrine actions and peripheral endocrine effects mediated through the bloodstream, gut hormones play a pivotal role relaying information on nutritional status to important appetite controlling centres within the central nervous system (CNS), such as the hypothalamus and the brainstem.

In this article, we will summarise our current understanding of the physiological interactions between the gut and brain, termed the “gut-brain axis,” focussing particularly on the interactions of gut hormones with the CNS and vagus nerve [6]. We will not discuss signal transduction pathways, enteric nervous systems related to controlling food intake, or neural signalling pathways in organs associated with the gastrointestinal tract such as liver or pancreas.

## 2. Gut Hormones

### 2.1. Pancreatic Polypeptide-Fold Peptides

The PP-fold family comprises neuropeptide Y (NPY), peptide YY (PYY), and pancreatic polypeptide (PP). They are composed of a chain of 36 amino acids residue and share amino acid homology, amidated C-terminal ends. The tertiary structure PP-fold is U shaped with an extended polyproline helix and an *α* helix connected by a *β* turn [7]. In addition, a hairpin-like PP-fold motif is vital for receptor binding. PYY and PP are secreted from gastrointestinal tract, whereas NPY is predominantly, widely distributed in CNS [8]. This family acts via G protein-coupled receptors; Y1, Y2, Y4, Y5, and Y6 [9].The Y3 receptor has not yet been cloned, and the Y5 receptor has been found as a nonfunctional truncated form.

### 2.2. Peptide Tyrosine Tyrosine (PYY)

PYY is an appetite suppressing hormone, which was isolated originally from porcine upper small intestine [8]. Its name is derived from its characteristic tyrosine (Y) residues at both the C and N terminals. PYY is released from the L cells of the distal gut in response to ingested nutrients with two other gut hormones, GLP-1 and OXM. PYY immunoreactivity is highest in the rectum, and decreases proximally to low levels in the duodenum and jejunum. PYY immunoreactivity is also found in the CNS regions such as the hypothalamus, medulla, pons, and spinal cord [10]. Two endogenous circulating forms, PYY_1–36_ and PYY_3–36_, are synthesized within the gut. PYY_1–36_ is the biologically active major circulating form, which is produced by cleavage of the N-terminal tyrosine-proline residues from PYY_1–36_ by the enzyme dipeptidyl-peptidase IV (DPP-IV) [11]. PYY_1–36_ has affinity to all Y receptors, while PYY_3–36_ acts mainly via the high-affinity hypothalamic Y2 receptor.

The PYY secretion pattern suggests a role in satiety. Circulating PYY concentrations are low in fasted state and increase rapidly following a meal with a peak at 1-2 hours and remain elevated for several hours [12]. PYY release is increased in proportion to calorie intake [12]. PYY may have a role in the pathogenesis of a number of anorectic conditions such as inflammatory bowel disease, steatorrhea, tropical sprue, and cardiac cachexia, since plasma PYY levels are elevated in patients with these conditions [13–15]. Peripheral PYY_3–36_ administration shows a reduction in food intake and body weight gain in rats [16]. In both lean and obese human subjects, intravenous administration of PYY_3–36_ reduces appetite and food intake [16, 17] with observed plasma PYY_3–36_ levels similar to the physiological levels after a meal; this data suggests that the physiological effect of PYY is to suppress food intake. Of note, no nausea was reported in subjects following PYY_3–36_ administration. This suggests that, unlike leptin, the sensitivity of subjects to PYY is preserved in obese subjects. Some investigators failed to show an anorectic effect of PYY, possibly due to inadequate acclimatization of control and treated animals [18].

The “ileal brake” is the negative feedback mechanism in which the presence of nutrients into the colon inhibits motility and transit of further nutrients within the upper gastrointestinal tract [19]. Fat is known to be the most potent trigger of the ileal brake. GLP-1 and PYY may contribute to this phenomenon [20].

PYY has been reported to regulate energy expenditure, delay gastric emptying, reduce acid secretion, and inhibit gallbladder contraction and pancreatic exocrine secretions [21, 22]. Circulating PYY levels are low in obese subjects [17, 23], and they are higher in patients with anorexia nervosa when compared with control subjects [24]. Studies of circulating levels of PYY in obese and lean people have yielded inconsistent results [25, 26]; however, a blunted postprandial rise in PYY in obese subjects suggests a possible association with impaired postprandial satiety during obesity [21].

PYY_3–36_ exerts anorectic effects via a direct action in the hypothalamic arcuate nucleus (ARC). Peripheral administration of PYY_3–36_ increases c-fos expression (a marker of neuronal activation) in the ARC and direct injection of PYY_3–36_ into the ARC inhibits food intake. This effect is likely to be mediated through the Y2 receptor since the anorectic effect of peripheral PYY_3–36_ administration is blocked in Y2 receptor-null mice, and intraarcuate injection of a Y2 receptor selective agonist also supresses food intake [16]. Although conflicting results have been reported [27], the vagal-brainstem may also signal the actions of PYY on food intake. Two independent laboratories have observed that vagotomy abolishes anorexia c-fos activation following peripheral PYY_3–36_ administration [28, 29].

In contrast to the anorectic effects observed by peripheral and intraarcuate PYY_3–36_ administration, direct administration of PYY_3–36_ into the third ventricle of the brain [30] or paraventricular nucleus (PVN) [31] increases in food intake. This paradoxical action may be explained by considering that such effects might be endogenously mediated by the orexigenic CNS-distributed peptide, NPY, through an action on Y1 receptor and Y5 receptors [32]. PYY may also act in the brain areas other than the hypothalamus and brainstem. In a clinical study using functional MRI by Batterham et al. [33], PYY_3–36_ infusion modulated neural activity within corticolimbic and higher cortical brain regions.

Exogenous PYY_3–36  NH2_ and exendin-4, a GLP-1 receptor agonist, have synergistic effects to suppress food intake in mice [34]. Furthermore a recent study utilizing functional MRI by De Silva et al. [35] showed that coadministration of PYY_3–36_ and GLP-1_7–36amide_ to fasted human subjects results in similar reductions in subsequent energy intake and brain activity, as observed physiologically following feeding.

Neuropeptide Y2 receptors have cardiovascular effects in addition to their metabolic effects. Y2 agonism is implicated in the pathogenesis of hypertension in hypertensive rats [36]. Nordheim and Hofbauer [37] reported that Y2 receptor stimulation by PYY_3–36_ demonstrated cardiovascular effects of endogenous NPY in rats on different dietary regimens. In food-restricted rats, PYY_3–36_ increased mean arterial pressure and heart rate, whereas PYY_3–36_ did not influence mean arterial pressure and heart rate in high-fat diet rats. However, human studies thus far have not demonstrated any hypertensive changes as a result of PYY administration.

### 2.3. Pancreatic Polypeptide (PP)

PP is secreted from PP cells in the pancreatic islets of Langerhans in response to a meal. Anorectic effects of PP are thought to be mediated by directly through the Y4 receptor in the brainstem and hypothalamus. In addition, it may act also via the vagus nerve, as the anorectic effects of PP are abolished by vagotomy in rodents [38]. PP has a high affinity for the Y4 receptor, of which expression is found in the area postrema (AP), nucleus of the tractus solitarius (NTS), dorsal motor nucleus of vagus (DVN), ARC, and PVN [39]. An autoradiography study also identified saturable PP binding sites at the interpeduncular nucleus, AP, NTS, and DVN [40]. Like PYY, paradoxical effects on food intake are observed following PP injection, depending on its route of administration. In contrast to the anorectic effects observed with peripheral PP administration, central PP administration stimulates food intake [41]. Although the exact mechanism of this phenomenon is unclear, these differential effects may be mediated by activation of distinct populations of receptors. PP also has other physiological effects, such as delaying gastric emptying, attenuating pancreatic exocrine secretion, and inhibiting gallbladder contraction [42].

Plasma PP levels show diurnal variations: lowest levels are observed in the early morning and highest in the evening. The release of postprandial PP is biphasic. Circulating PP concentrations increase after a meal in proportion to the caloric intake, and increased levels remain for up to 6 hours postprandially [43]. Circulating PP levels seem to be inversely proportional to adiposity; higher levels are reported in subjects with anorexia nervosa [44]. Some, but not all [45, 46], studies have demonstrated significant reductions in circulating levels of PP in obese subjects [47, 48]. Furthermore, obese patients with Prader-Willi syndrome (PWS) have been reported to have reduced PP release both basally and postprandially [49].

In mice, acute and chronic peripheral PP administration results in reduced food intake. In leptin-deficient *ob/ob* mice, repeated intraperitoneal PP injection decreases body weight gain and improves insulin resistance and hyperlipidaemia [38]. Furthermore, transgenic mice overexpressing PP have reduced food intake when compared with wild-type controls [50]. In normal-weight human subjects, intravenous infusion of PP achieved three times higher circulating PP concentrations when compared with postprandial levels in the same subjects after a buffet lunch (which reduced food intake by 25% over 24 hours) [51]. Furthermore, twice-daily infusion of PP in volunteers with PWS resulted in a 12% reduction in food intake [52]. Agonists to the Y4 receptor designed to mimic the actions of PP have been developed and are under further investigation as potential novel therapies for obesity.

### 2.4. Proglucagon-Derived Peptides

The proglucagon gene is expressed in the pancreas, in the L-cells of the small intestine and in the NTS of the brainstem [53, 54]. GLP-1, GLP-2, OXM, and glucagon are proglucagon-derived peptides. Glucagon is the main product in the pancreas, whereas OXM, GLP-1, and GLP-2 are the major products in the brain and intestine [55].

#### 2.4.1. Glucagon-Like Peptide-1 (GLP-1)

GLP-1 is cosecreted with PYY from the L cells in the intestine in response to nutrient ingestion. GLP-1 has two biologically active forms, GLP-1_7–37_ and GLP-1_7–36amide_. The latter truncated form is the major circulating form in humans, although both active isoforms of GLP-1 have equivalent potency [56]. In addition, GLP-1_7–36amide_ is distributed within the CNS. Immunoreactive neurons for GLP-1_7–36amide_ are located in the PVN, DMN, NTS, dorsal vagal complex (DVC), pituitary, and thalamus [57]. GLP-1 receptor mRNA is distributed throughout the rostrocaudal hypothalamus, with dense accumulation in the ARC, PVN, and supraoptic nuclei [58]. While peripheral administration of GLP-1 in rats leads to increased c-fos expression in the ARC [28], intracerebroventricular (ICV) administration results in increased c-fos expression in the PVN, NTS, and AP [59]. Ascending NTS-PVN projections contain GLP-1 [60] are implicated in controlling food intake. In the CNS, leptin receptor (Ob-Rb) was expressed in GLP-1-containing neurons in the NTS in animals and leptin activated GLP-1 containing neurons in the NTS [61]. Signals arising from the hepatoportal GLP-1R promote glucose clearance, which are independent of changes in insulin secretion [62, 63].

 GLP-1 exerts its effect by activation of the GLP-1R to stimulate *adenylyl cyclase* activity and thereby *cAMP* production [64]. GLP-1R is widely distributed particularly in the brain, gastrointestinal tract, and pancreas [64, 65]. In the brain, binding sites for GLP-1Rs have been found in the hypothalamus, striatum, brainstem, substantia nigra, and subventricular zone among other structures [64, 66]. GLP-1Rs are present on both glia and neuronal cell types [66]. In addition, GLP-1Rs are expressed in the nodose ganglion [67]. Furthermore bilateral subdiaphragmatic total truncal vagotomy or brainstem-hypothalamic pathway transetioning abolishes the suppressing actions of GLP-1 on food intake [28]; this suggests that the vagus contributes to the actions of GLP-1 on food intake.

Circulating GLP-1 levels rise postprandially and fall in the fasted state. Recent evidence also suggests that GLP-1 levels rise in anticipation of a meal [68]. GLP-1 not only reduces food intake, but also suppresses glucagon secretion and delays gastric emptying [69]. Intravenous administration of GLP-1 is associated with a dose-dependent reduction of food intake in both normal weight and obese subjects [70], although obese subjects may be less responsive [64].

GLP-1 possesses a potent incretin effect in addition to its anorectic action; it stimulates insulin secretion in a glucose-dependent manner following ingestion of carbohydrate. However, its use as obesity treatment was limited for many years by its short plasma half-life of 1-2 minutes [71], which is partly attributed to enzymatic degradation by DPP-IV and renal clearance that rapidly inactivate and remove GLP-1 from plasma circulation [72, 73]. Continuous subcutaneous infusion of GLP-1 to patients with type 2 diabetes for 6 weeks reduces appetite, and body weight, and improves glycaemic control [74]. However, DPP-IV-resistant analogues of GLP-1 have been developed. Exendin-4 (exenatide), a naturally occurring peptide originally isolated from the saliva of the Gila monster lizard, is a DPP-IV-resistant GLP-1R agonist [75]. Exenatide improves glycaemic control and decreases body weight in patients with type 2 diabetes. [76]. GLP-1 possesses trophic effects on pancreatic beta cells in animal models [77]. GLP-1 and exendin-4 have been recently shown to promote cellular growth and reduce apoptosis in nervous tissues [78], but trophic effects on pancreatic beta cells have not been demonstrated clinically in human subjects. GLP-1 agonists are, therefore, a good example of how research in this area has been translated into clinical practice. A three-year duration of treatment with exenatide has been reported to improve beta cell function; however, when adjusting for weight loss associated with exenatide therapy, this effect remains speculative [79]. DPP-IV inhibitors, such as sitagliptin and vildagliptin, which are licensed for the treatment of type 2 diabetes, do not result in decrease in body weight. This may be explained by considering that DPPIV is also involved in the modification of other gut hormones such as PYY, and cytokines which may have opposite effects to GLP-1 [80].

GLP-1-based therapies are promising novel treatments for type 2 diabetes, however, long-term outcome data are not yet available. The reported side effects of GLP-1 agonists are nausea and vomiting. Animal safety studies with liraglutide have identified C-cell carcinoma of the thyroid. Acute pancreatitis has been reported in humans treated with liraglutide or exenatide [81]. Further outcome data will, therefore, be important in confirming the long-term safety of GLP-1-based therapies.

#### 2.4.2. Oxyntomodulin (OXM)

OXM is a 37-amino acid peptide originally isolated from porcine jejunoileal cells and is found to show glucagon-like activity in the liver [82]. OXM is another product of the proglucagon gene and is cosecreted with GLP-1 and PYY by the L-cells of the distal gastrointestinal tract, in response to ingested food and in proportion to caloric intake [83]. OXM has anorectic effects and shows incretin activity with a much lower potency when compared with GLP-1 [84]. OXM also inhibits gastric acid secretion and delays in gastric emptying [85].

Administration of OXM is associated with decreased food intake and increases energy expenditure in both rodents and humans [86–88]. The anorectic effect of OXM is blocked by the GLP-1R antagonist, exendin_9–39_ [89], and is not observed in GLP-1R null mice [90]; this suggests that the anorectic effects of OXM may be mediated by the GLP-1R. However, OXM has relatively low *in vitro *affinity for the GLP-1R which is 50 folds lower than the affinity of GLP-1 for GLP1R, despite the anorectic potency of OXM being comparable to the potency of GLP-1 [91]. Several actions of OXM seem independent of the GLP-1R [87, 92, 93]; the cardiovascular effects of OXM are preserved in GLP-1R knockout mice [92]. These data suggest that a further receptor through which OXM mediates its anorectic effect has yet to be identified. Furthermore, direct administration of the GLP-1R antagonist, exendin_9–39_, to the ARC fails to inhibit the anorectic effects of OXM but inhibits that of GLP-1 [87]. Like GLP-1, OXM is inactivated by DPP-IV. OXM analogues resistant to DPP-IV degradation are being developed as potential obesity treatments [94].

#### 2.4.3. Glucagon

The role of glucagon in glucose homeostasis is well established; glucagon is produced by alpha cells of the pancreatic islets and increases glucose concentration in response to hypoglycaemia. Glucagon enhances the body's physiological response to stress, by increasing energy expenditure [95, 96]. However, glucagon administration also decreases food intake, possibly by modulating vagal tone and gastric emptying [97, 98]. Schulman et al. [99] reported that glucagon reduces food intake and body weight but caused hyperglycemia. However, the administration of the dual agonists stimulating both glucagon and GLP-1 receptors achieved improvement of diet-induced obesity and glucose intolerance [100, 101]. It is, therefore, plausible that dual agonism of glucagon and GLP-1 receptors may offer novel targets for antiobesity treatment.

### 2.5. Ghrelin

Ghrelin was identified originally as an endogenous ligand for the growth hormone secretagogue receptor (GHS-R) in rat stomach [102]. Ghrelin comprises a chain of 28 amino acids with esterification of the hydroxyl group of the third serine residue by octanoic acid, and it is the only known orexigenic gut hormone. Ghrelin is principally secreted from X/A-like cells within gastric oxyntic glands [103]. In keeping with this, gastrectomy results in an 80% reduction of plasma ghrelin levels; the remainder is secreted from the intestine, pancreas, pituitary, and colon [104]. Ghrelin also acts as a neurotransmitter, being expressed within the ARC and periventricular area of the hypothalamus [102, 105].

Serum ghrelin levels are increased by fasting and decreased by refeeding or oral glucose administration, but they are not decreased by water ingestion [106]. In rats, ghrelin levels show a diurnal pattern, with the bimodal peaks occurring before dark and light periods [107]. In humans, ghrelin levels have a diurnal rhythm which is identical to the diurnal rhythm of leptin, with both hormones rising throughout the day to a zenith at 0100 h, then falling overnight to a nadir at 0900 h [108].

Levels of circulating ghrelin rise preprandially and fall rapidly in the postprandial period [108]. Both central and peripheral administration of ghrelin increase food intake and body weight along with a reduction in fat utilisation in rodents [106, 109]. Negative correlations between circulating ghrelin levels and body mass index are found in human. Fasting plasma levels of ghrelin are reported to be high in patients with anorexia nervosa [110] and subjects with diet-induced weight loss [111]. In contrast, obese subjects show a less marked drop in plasma ghrelin after meal ingestion [112]. In patients with heart failure, increased levels of plasma ghrelin are reported in cachectic patients when compared with noncachectic patients [113]. Furthermore, in patients with PWS, elevated circulating ghrelin levels are found, when compared with individuals with nonsyndromic forms of obesity [114].

Ghrelin mediates its orexigenic action via stimulation of NPY/agouti-related peptide (AgRP) coexpressing neurons within the ARC of hypothalamus. Peripheral administration of ghrelin increases c-fos expression in the ARC NPY/AgRP neurons [115] and ablation of both AgRP and NPY neurons completely abolishes the orexigenic effect of ghrelin [116]. The brainstem and vagus nerve may also contribute to the effects of ghrelin on food intake. ICV injection of ghrelin induces c-fos expression in the NTS and AP [117]. GHS-R is found to be expressed in the vagus nerve. Furthermore, blockade of gastric vagal afferents in rats abolishes ghrelin-induced feeding and prevents the ghrelin-induced rise in c-fos expression within the ARC [118]. In addition to its potent orexigenic property, ghrelin also increases gastric motility, upstimulates the hypothalamo-pituitary-adrenal axis, and possesses cardiovascular effects such as vasodilatation and enhanced cardiac contractility [104].

Ghrelin may promote food intake in part by enhancing the hedonic responses to food cues, which is demonstrated by the recent study by Malik et al. [119]. In their study, functional MRI was performed during exposure to food pictures, and the study results demonstrated increased activation in the amygdala, orbitofrontal cortex, anterior insula, and striatum, during intravenous infusion of ghrelin.

### 2.6. Obestatin

Obestatin is a 23-amino acid peptide hormone which is derived from posttranslational cleavage of preproghrelin, and released from the stomach [120]. In contrast to ghrelin which has orexigenic properties, obestatin may have anorectic effects by decreasing food intake, delaying gastric emptying, and reducing body weight in rodents [121]. However, the potential anorectic of obestatin remains controversial, since other investigators have failed to demonstrate effects on food intake in lean or obese rodents [122].

### 2.7. Cholecystokinin (CCK)

CCK was the first gut hormone found to be implicated in appetite control [123]. CCK is secreted postprandially by the I cell of the small intestine into circulation [124], with a short plasma half-life of a few minutes. Plasma CCK levels rise within 15 minutes after meal ingestion [124]. Infusion of C-terminal octapeptide of CCK decreased food intake in 12 lean men [125]. However, intermittent prandial CCK infusion reduces meal size in rats but causes a compensatory increase in meal frequency [126]. A 2-week continuous intraperitoneal infusion of CCK failed to suppress food intake at any time point [127]. Other physiological functions of CCK include stimulating the release of enzymes from the pancreas and gall bladder, promoting intestinal motility, and delaying gastric emptying. There are two CCK receptor subtypes known; CCK1 and CCK2 receptors, previously classified as CCK A and CCK B. The anorectic action of CCK appears to be mostly mediated via CCK1 receptors on the vagal nerve [128, 129]. CCK 1 and 2 receptors are widely distributed in brain including the brainstem and hypothalamus [130].

Some studies suggest that leptin and CCK may interact synergistically to induce short-term inhibition of food intake and long-term reduction of body weight [131]. Leptin-deficient mice are insensitive to the meal-terminating effect of CCK administration. Furthermore, leptin signalling pathways to brain are dampened in the absence of interaction with CCK release after a meal or in the setting of CCK-A receptor blockade [132].

### 2.8. Amylin

Amylin is *coreleased* with insulin in response to meal ingestion, and it may function as an anorectic hormone. Circulating levels of amylin are found to be higher in obese than lean subjects [133, 134]. Administration of amylin is associated with reduced food intake and body weight [135]. The anorectic effects of amylin may be mediated by modulating activity of the serotonin, histamine, and dopaminergic system in the brain as well as inhibition of NPY release [133]. Administration of pramlintide, a synthetic analogue of human amylin, improves glycaemic control and causes weight loss in type 2 diabetes patients using insulin [136]. Therefore, amylin replacement with pramlintide as an adjunct to insulin has been reported as a novel physiological approach toward improved long-term glycaemic and body weight control in patients with diabetes [137].

## 3. Peripheral Adiposity Signals

### 3.1. Insulin

Circulating levels of insulin and leptin positively correlate with adipose tissue mass within the body. Both insulin and leptin are implicated in the long-term regulation of energy balance. Insulin is synthesized in the ß cells of the pancreas and is secreted rapidly after a meal, with well-characterised hypoglycaemic effects [138]. However, insulin also acts as an anorectic signal within the CNS. ICV administration of insulin results in a dose-dependent suppression of food intake and body weight gain in baboons and rodents [139, 140]. Intrahypothalamic insulin injection to the PVN also results in decreased food intake [141]. Insulin enters the CNS through a saturable and receptor-mediated transport process [142]. Insulin receptors are widely expressed in the brain, particularly in hypothalamic nuclei, such as the ARC, DMN, and PVN, which are involved in control of food intake [143]. Although the mechanism of insulin-mediated anorexia has not been fully elucidated, hypothalamic NPY seems to be involved. ICV administration of insulin inhibits the fasting-induced increase in NPY mRNA expression in the PVN and ARC in rats. This suggests that fasting increases NPY biosynthesis through an ARC-PVN pathway in the hypothalamus via a mechanism which is dependent on low insulin levels [144].

### 3.2. Leptin

Leptin is the product of the *ob* gene, and it is predominantly secreted by adipocytes with circulating levels proportional to fat mass [145]. Levels of circulating leptin have a diurnal and pulsatile pattern, with peak levels at night [146]. Leptin is transported across the BBB by a saturable transporter system [147], and it exerts its anorectic effect via the ARC, where both NPY/AgRP and pro-opiomelanocortin (POMC)/cocaine- and amphetamine-regulated transcript (CART) neurons express leptin receptors [148]. Leptin inhibits NPY/AgRP neurons and activates POMC/CART neurons [149, 150], resulting in reduced food intake [149] and increased energy expenditure [151]. The effects of gut satiation signals such as CCK can be amplified by leptin which acts in the CNS, including the ARC in particular [152].

There are three types of leptin receptors identified: long, short, and secreted form [153]. Among those, Ob-Rb receptor, which is highly expressed in the hypothalamus [154], is thought to act as the main receptor involved in appetite control. The *db/db* mouse, with an inactivating mutation in the Ob-Rb receptor, has an obese phenotype [155, 156], and leptin-deficient *ob/ob* mice exhibit hyperphagia and obesity, which can be reversed by leptin administration [157].

Subcutaneous administration of recombinant leptin reduces fat mass, hyperinsulinaemia, and hyperlipidaemia in obese children with congenital leptin deficiency [158]. However, obese individuals often have high leptin levels, which result in a failure to respond to exogenous leptin. This leptin resistance severely limits the therapeutic utility of leptin, and it is likely to result from reduced leptin receptor signal transduction [159] or an impaired ability of the BBB to transport leptin [160].

## 4. Neural Pathways Related to the Appetite Control

Feeding and energy expenditure are controlled by complex neural networks distributed throughout the forebrain and brainstem. Reward-related neural brain regions have been implicated in the nonhomeostatic control of feeding behaviour [161]. By contrast, homeostatic feeding behaviour is integrated within the hypothalamus. Key peripheral signals of energy status such as gut hormones and adipokines either signal to the hypothalamus directly or signal to the hypothalamus indirectly via the brainstem and vagal afferent fibres. Adiposity signals such as insulin and leptin are involved in the long-term energy homeostasis, and gut hormones such as PYY, GLP-1, PP, OXM, and ghrelin are implicated in the short-term regulation of meal ingestion [162–164].

## 5. The Hypothalamus

The hypothalamus controls feeding by integrating peripheral humoral signals that influence food intake and energy expenditure, with neural signals from the brainstem and higher cortical centres. The importance of the hypothalamus in energy homeostasis was first suggested by classic lesioning experiments in rodents [165]; subsequent studies have suggested a role of hypothalamic nuclei, such as arcuate nucleus (ARC), paraventricular nucleus (PVN), ventromedial nucleus (VMN), dorsomedial nucleus (DMN), and lateral hypothalamic area (LHA) in energy homeostasis.

**Figure 1:**
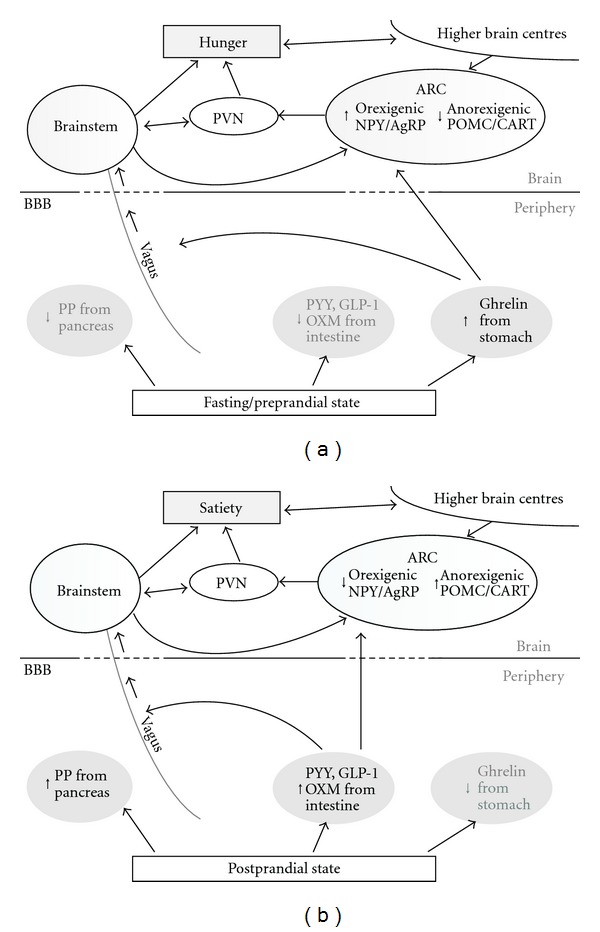
The gut hormone signalling to the brain under fasted (a) and fed states (b). (a) During the fasting/preprandial state, ghrelin release from the stomach acts upon the ARC and vagus to stimulate hunger. (b) In the postprandial state, release of anorectic hormones, PYY, GLP-1, OXM, and PP from intestine act upon the ARC, brainstem, and vagus to cause satiety. ARC, arcuate nucleus; NPY/AgRP, neuropeptide Y and agouti related peptide; POMC/CART, pro-opiomelanocortin, and cocaine- and amphetamine-regulated transcript; PVN, paraventricular nucleus; GLP-1, glucagon-like peptide-1; PP, pancreatic polypeptide; PYY, peptide YY; OXM, oxyntomodulin.

In the ARC, there are two important discrete neuronal populations: neurons which coexpress neuropeptide Y (NPY) and agouti-related peptide (AgRP) stimulate food intake, whereas neurons coexpressing pro-opiomelanocortin (POMC) and cocaine- and amphetamine-regulated transcript (CART) suppress food intake (Figure 1). The ARC is located at the base of median eminence which may be accessed by circulating hormones likely due to its deficient blood-brain barrier (BBB) [166] or by carrier-mediated transport.

Cleavage of a precursor protein, called POMC, produces *α*-melanocyte-stimulating hormone (*α*-MSH), which binds to melanocortin-3 receptor (MC3R) and melanocortin-4 receptor (MC4R) to suppress food intake [167]. The MC4R is highly expressed in the hypothalamus and is thought to have a major role in suppressing food intake compared to the MC3R. MC4R knock-out mice have hyperphagia and obesity [167]. MC3R-deficient mice also have increased fat mass and reduced lean body mass [168]; however, selective MC3R agonists fail to suppress feeding [169].

CART is the third most abundant transcript identified within the hypothalamus and is mostly colocalized with POMC in the ARC. ICV administration of CART suppresses feeding, whereas ICV injection of CART antiserum increases food intake [170]. However, CART injected directly into the PVN or ARC of fasted rats causes an increase in food intake at 1-2 hours postinjection [171], which suggests opposing effects of CART on food intake can be observed depending on the site of administration. Hence, the physiological role of CART in energy homeostasis is unclear.

NPY/AgRP neurons extensively project to the adjacent hypothalamic nuclei, such as the PVN, DMN, and LHA. AgRP and NPY are exclusively colocalized in ARC neurons, both of which exert orexigenic effects [172]. NPY is the most abundant neuropeptide in the CNS [173]. ICV injection of NPY stimulates food intake in rats [41] and repeated daily bilateral PVN injection of NPY for 10 days causes an approximately two-fold increase in daily food intake and a six-fold increase in the rate of body weight gain [174]. The orexigenic effect of NPY appears to be mediated by stimulation of hypothalamic Y1 and Y5 receptors. AGRP was isolated by its high-sequence homology with the Agouti coat colour gene which is associated with yellow coat, obesity, and increased body length in mice. AgRP is a potent-selective antagonist at the MC3R and MC4R [175].

The PVN receives projections of NPY/AgRP and POMC/CART from the ARC and contains neurons which express the anorectic factors, thyrotropin-releasing hormone, and corticotropin-releasing hormone. Microinjection of orexigenic or anorexigenic signals, such as ghrelin, orexin-A, CCK, leptin, and GLP-1 into the PVN alter food intake and body weight [163]. While ICV injection of NPY into the PVN causes hyperphagia and obesity [174], destruction of the PVN causes hyperphagia and obesity [176]. This finding implies that the PVN may have an inhibitory role in food intake and body weight. The LHA also receives projections from the ARC and contains two orexigenic neuropeptides, melanin-concentrating hormone (MCH), and orexin (hypocretin). Transgenic mice overexpressing MCH develop obesity and insulin resistance [177]. Furthermore, transgenic mice which are deficient in the prohormone precursor of MCH or the MCH 1 receptor are lean [178]. Hypocretins 1 and 2 are produced by the groups of neurons in the LHA [179]. These neurons project extensively to the olfactory bulb, cerebral cortex, thalamus, hypothalamus, brainstem, locus coeruleus, tuberomamillary nucleus, and raphe nucleus. In addition to the orexigenic effects on food intake, orexin seems to also have a role in other physiological functions such as regulation of blood pressure, the neuroendocrine system, body temperature, and the sleep-waking cycle [180]. An impairment of hypocretin neurotransmission has been associated with the pathology of human narcolepsy, which is a chronic sleep disorder characterized by excessive daytime sleepiness, cataplexy, hypnagogic hallucinations, and sleep paralysis [181]. MCH-R1 antagonists may have therapeutic potential for the treatment of obesity [182], but further work is required to determine if their use would be associated with adverse effects attributable to the other biological actions of orexin.

The DMN receives NPY/AgRP projections from the ARC [183] and projects the *α*-MSH fibre to the PVN [162, 184]. DMN lesions cause hyperphagia and obesity, which suggests a suppressive role in appetite [185]. In diet-induced mice, an approximately 40-fold increase in NPY expression is observed in the DMN and VMN when compared with controls [186]. In the VMN, brain-derived neurotrophic factor (BDNF) is highly expressed, and VMN BDNF neurons suppress food intake through MC4R signalling [187]. Increased signalling in the VMN following an oral glucose load has been observed [162]. Selective deletion of BDNF neurons in the VMN and DMN of adult mice results in hyperphagia and obesity [188].

Glucose sensing plays an important role of the brain. Conventionally, glucose sensing is thought to involve glucokinase-dependent metabolism of glucose to ATP, which then alters membrane excitability by modulating ATP-dependent channels or transporters, such as ATP-inhibited K+ channels (KATP). Recent studies, however, suggest that glucose-excited and glucose-inhibited neurones are able to sense glucose irrespective of such metabolic pathways. Brain glucose sensors, specialized neurones which respond to fluctuations in local extracellular glucose concentration, have been found only in a few brain regions, in particular, the hypothalamus and brainstem. Hypothalamic glucose-sensing neurones are found in the LHA, ARC, and VMN, and responsive neurons have been identified which either increase firing rate (glucose-excited neurones) or decrease firing rate (glucose-inhibited neurones) in response to extracellular glucose [189].

## 6. Brainstem

Within the brainstem, the dorsal vagal complex (DVC) plays an important role in relaying peripheral signals via vagal afferent fibres from the gut to hypothalamus. The DVC has projections to the hypothalamus and higher cortical centres [190] and comprises the dorsal motor nucleus of vagus (DVN), area postrema (AP), and the nucleus of the tractus solitarius (NTS). NTS is an ideal position to integrate peripheral signals due to its close proximity to the AP, which has an incomplete BBB [163].

Other than ascending brainstem-hypothalamus pathways, descending hypothalamic projections to the brainstem are also important in control of food intake. *α*-MSH projections from POMC neurons in the ARC terminate in close anatomical proximity to neurons in the NTS, which respond to gastric distension [191]. Furthermore, descending projections from the LHA to the NTS contain orexin and MCH, and descending ARC-parabrachial nucleus pathways have been identified [152]. The PVN projects regions of the midbrain such as the ventral tegmental area, Edinger-Westphal nucleus, ventrolateral periaqueductal gray matter, reticular formation, pedunculopontine tegmental nucleus, and dorsal raphe nucleus. The PVN also projects to the prelocus coeruleus in the dorsal pons as well as the nucleus ambiguous and NTS in the ventral medulla. The medial NTS receives the most extensive projections of the PVN, substantially more than the DVN or AP [192].

The importance of the hindbrain in energy homeostasis is highlighted by considering that chronically maintained decerebrate rats, with complete high mesencephalic transection, remain responsive to taste stimuli and respond to intake-inhibitory feedback from the gut; however, hyperphagic response to food deprivation is not observed in these animals [193]. Direct delivery of leptin to the lateral or third ventricle as well as the fourth ventricle significantly suppresses food intake up to 24 h after treatment [193]. The effects of various gut hormones on food intake are attenuated by lesions of the area postrema [194] or vagotomy [28, 29, 118, 195]. Taken together, these findings suggest brainstem-mediated mechanisms on controlling food intake.

The expression of leptin and insulin receptors, and of glucose sensing mechanisms in the brainstem, is similar to that seen in the hypothalamus [193]. POMC neurons exist within the NTS, which show STAT-3 activation in response to leptin administration to suppress food intake [196]. Administration of leptin into the DVC suppresses food intake [193].

In addition to the hypothalamus, the vagus nerve plays a central role in regulating the feeding. Vagal afferent neurons have been shown to express a variety of receptors within the brainstem, which include cholecystokinin (CCK) 1R and CCK2R (at which both CCK and gastrin act [197]), Ob-R [198], Y2R [29], GLP-1 [67], and GLP-2R [199], growth hormone secretagogue receptor (GHS)-R1 where ghrelin acts [118], and the orexin receptor, OX-R1 [200].

The vagal stretch and tension sensors detect nutrients stored in the stomach. The vagus nerve also helps to transmit gut hormones signals such as CCK, ghrelin, PYY, PP, and GLP-1, which are released by anticipation of meals and the presence of food in the upper gastrointestinal tract. Cell bodies of afferent fibres of the abdominal vagus nerve are located in the nodose ganglia, which project to the DVC of brainstem. In rats, infusion of saline into the stomach has been observed to reduce food intake to similar extents to infusion of nutrients into the stomach [201]; this phenomenon is likely to be attributable to vagal nerve function. The vagus nerve participates in transmitting the food-induced negative-feedback signals important for determining meal size. Transection of all gut sensory vagal fibres results in increased meal size and meal duration, but does not block gastric preload-induced feeding suppression, implying that vagal afferent signals have a significant role in satiety during spontaneous meals [202, 203]. Randich et al. [204] utilized extracellular recordings from the vagus nerve, and found that it transmits a satiety signal from the jejunum, following activation by infusion of fatty acids.

The importance of the role of the vagus nerve in transmitting peripheral signals has been demonstrated by vagotomy or capsaicin treatment to abolish its effect, and by vagus nerve stimulation (VNS) to enhance its activity [205]. Low-frequency VNS in rats fed with a standard diet results in decreased food intake and body weight [206]. Compared with the sham group, obese minipigs received VNS did not gain body weight and showed decreased food intake by 18%—the effects lasted for 14 weeks [207]. Gil et al. [208] reported that chronic VNS with 10 Hz electrical impulses in rats fed with a high-fat diet significantly decreased food intake and body weight gain. In their study, significant neuronal responses in the NTS and decreased serum leptin, but increased ghrelin levels, were observed and also nesfatin-1 levels tended to increase following VNS. This suggests that VNS results in reductions in food intake and body weight by increasing brain satiety signals through the vagal afferents.

The close association of the vagal efferent, sympathetic, and enteric systems makes it difficult to selectively manipulate the vagus nerve. Genetic approaches to modulate signalling of neurotrophin factors (e.g., BDNF and neurotrophin-3), which are essential for vagal afferent development, may help to further elucidate the regulatory role of the vagus nerve in gut physiology [209].

## 7. Reward Systems

In humans, environmental cues, cognitive, reward, and emotional factors play an important role in food intake which may override homeostatic requirements [210]. The corticolimbic pathways are responsible for reward-associated feeding behaviour, which include the striatum, ventral tegmental area, nucleus accumbens, insular cortex, anterior cingulate cortex, and orbitofrontal cortex. The orbitofrontal cortex is associated with regulating gustatory, olfactory, visual, and somatosensory function, and sensory factors, such as taste and smell, and has an important role in reward-related feeding [211]. In patients with frontotemporal lobar degeneration, hyperphagia is reported to be associated with atrophy in the anterolateral orbitofrontal cortex [212].

The endocannabinoid and opioid systems have wide receptor distributions within the CNS and play important roles in reward-related feeding [213]. Administration of a *μ*-opioid receptor agonist into the nucleus accumbens preferentially stimulates intake of high-fat diet regardless of diet preference at baseline, when both fat and carbohydrate diets are displayed simultaneously [214]. Increased expression of orexin in the hypothalamus has been observed following administration of opioid *μ*-receptor agonists into the nucleus accumbens [215]. Preadministration of a cannabinoid receptor (CB1) antagonist prevents the orexigenic effect of the endocannabinoid agonist, anandamide on food intake [213]. Leptin has been shown to reduce endocannabinoid levels in the hypothalamus [216]. This suggests that hypothalamic endocannabinoids may act via CB1 to increase food intake through a leptin-regulated mechanism. The nucleus accumbens (NAs) is a key region of limbic pathway and may be implicated in regulation of hedonistic feeding and homeostatic feeding [210].

The ventral striatum and population of dopamine neurons of the substantia nigra are involved in the reward system in human and nonhuman primates. The ventral striatum receives input from the orbitofrontal cortex and anterior cingulate cortex, which include the NA and the broad continuity between the caudate nucleus and the putamen and the olfactory tubercle [217]. Dopamine appears to be associated with reward-related food intake and with behaviours required to maintain feeding essential for survival. Mice lacking dopamine, caused by the selective inactivation of tyrosine hydroxylase, develop fatal hypophagia; and replacement of dopamine in these animals to the caudate putamen or NA restores preference for sucrose or palatable chow [218]. However, dopamine may have more complex effects on feeding, since dopamine signalling in the DMN and ARC of hypothalamus may inhibit food intake [149].

In a recent positron emission tomography study, meal ingestion was associated with greater activation of the midbrain and middle-dorsal insula, and lesser activation in the posterior cingulate cortex, temporal cortex, and orbitofrontal cortex following a meal in obese individuals when compared with lean individuals [219]. In addition, a study utilizing functional magnetic resonance imaging (MRI) suggested that obese individuals had greater responses to odours and fat rich food when compared with lean subjects [220].

## 8. Nutrients and its Related Signals Modulating Appetite

Although low-energy density diets induce short-term weight loss [221], they are usually associated with rebound weight gain. Some dietary patterns such as the Mediterranean-type diet are associated with a decreased rate of cardiac death and nonfatal myocardial infarction compared with a Northern European or North American dietary pattern [222]. In the Mediterranean diet, monounsaturated fat is substituted for saturated and trans-fats, and intake of fruits, vegetables, fibre, and whole grains are high, is recommended [223]. It is, therefore, interesting to consider whether specific nutrients within diets are able to modulate food intake, in addition to effects associated with their direct nutritional value.

The amino acid L-Glutamate is involved in multiple physiologic functions, which include taste perception, carbohydrate metabolism, and excitatory neurotransmission in the brain [224]. L-Glutamate stimulates its receptors in gut epithelial cells, which activate cerebral regions such as basal ganglia, limbic systems, and hypothalamus through vagal afferent nerve [225]. Kondoh et al. [226] reported that rats with chronic *ad libitum* administration of monosodium L-glutamate had reduced weight gain, fat deposition, and plasma leptin concentrations when compared with controls.

In rodents, long-chain omega-3 polyunsaturated fatty acids supplementation has been shown to improve obesity [227]. In a recent study using a mouse model of diet-induced obesity, ICV administration of unsaturated fatty acids reduced hypothalamic inflammation, hypothalamic and whole body insulin resistance, and body adiposity [228]. Free fatty acids exert insulin-like effects in key brain areas for energy homeostasis, including the ARC, possibly by favouring intracellular accumulation of the long-chain fatty acyl-CoA (LCFA-CoA) [229].

Unsaturated free fatty acid may, therefore, be beneficial to treat obesity, although evidence in human is still limited.

Fructose is being increasingly used in processed foods within the Western diet. However, its effects may be distinct to those of the related sugar, glucose. As glucose levels entering to the brain increase, food intake is suppressed. In contrast, fructose increases food intake when metabolized in the brain. Fructose has the opposite effect of glucose on the AMP activated kinase/malonyl-CoA signaling system and thereby enhances feeding behaviour [230].

Serotonin has a role in appetite control. 5HT-containing neurons are organized into nine nuclei (B1–B9) which are located in the midbrain and hindbrain. In particular, the midbrain dorsal raphe (B7) contains a substantial portion of the total brain 5HT neurons, with distinct projections to hypothalamic nuclei and other feeding-related forebrain areas [231]. 5-HT-stimulating drugs reduce food intake partly mediated through the 5-HT_2C_ receptor [232]. Although its effects on eating behaviour remain to be characterised, lorcaserin, a selective 5-HT_2C_ receptor agonist is reported to be a novel antiobesity drug reducing both food intake and body weight.

Taste affects food preference and intake. Lingual proteins CD36 and GPR120 are reported to be responsible for the spontaneous preference for lipid-rich foods [233] and have been identified in human taste buds [234]. The gut hormones such as GLP-1 and CCK and neurotransmitters are also produced locally in taste buds [235]. Sweet and umami taste are mediated by T1R family (T1R1, T1R2, T1R3) which belongs to family C of GPCRs including metabotropic glutamate receptors, calcium sensing receptors, and V2r pheromone receptors. In the intestine, there are different sweet taste cells (enteroendocrine, brush cells) within the epithelial layer. These sweet taste receptors may signal through vagal afferent fibres to alter food intake and delay gastric emptying [236]. It has been shown that leptin selectively suppresses sweet taste sensitivity or taste cells through Ob-RB, whereas endocannabinoids enhance sweet taste sensitivity of taste cells via CB1 receptor [237].

## 9. Bariatric Surgery

Whereas pharmacological and behavioural treatments are usually associated with weight loss followed by weight regain, bariatric surgery provides weight loss for at least 15 years, in patients with obesity [238, 239]. Bariatric surgery is classified into 3 types of surgical procedures; malabsorptive surgery, restrictive surgery, and mixed procedures. Malabsorption-based procedures include the jejuno-ileal bypass, which results in decreased nutrients absorption by shortening the functional small bowel length, and by allowing nutrients to pass directly from the proximal jejunum to the terminal ileum. Restrictive bariatric surgery includes the laparoscopic application of an adjustable gastric band, which is associated with lower comorbidity when compared with malabsorption-based procedures [240]. Roux-en-Y gastric bypass (RYGB) is a combined restrictive and malabsorptive procedure, which yields long-term, sustained period of weight loss with an acceptable level of risk. RYGB is thought to achieve its beneficial effects through the BRAVE effects: Bile flow alteration, Reduction of gastric size, Anatomical gut rearrangement and altered flow of nutrients, Vagal manipulation, and subsequent Enteric gut hormone modulation [241]. A decrease in levels of the orexigenic hormone ghrelin, and an increase in levels of the anorectic hormones PYY and GLP-1 have been observed following bypass surgery [111, 242, 243]. An increase in energy expenditure may play a role in part in weight loss after gastric bypass surgery [244]. Moreover, microbial shifts towards substantially higher concentrations of *Proteobaceria*, specifically *Enterobacter hormaechei*, are demonstrated following RYGB surgery [241]. Gastric bypass surgery is associated with greater improvements in glycaemic control in patients with type 2 diabetes, when compared with gastric banding procedures [245]. Furthermore these improvements in glycaemic status often precede weight loss, which implies that bypass surgery may have effects in ameliorating type 2 diabetes which are additional to their effects on body weight.

## 10. Gut Microbiota

A potential association between gut microbiota and the pathogenesis of obesity has been recently recognised [246]. The human gut harbours a large number of 1000 to 1150 bacterial species collectively termed gut microbiota [247]. Adult germ-free mice have 40% less total body fat than mice with normal microbiota; and replacing the microbiota in adult germ-free mice is associated with a 60% increase in body fat content and insulin resistance within 14 days of replacement [248]. In contrast to mice with normal gut microbiota, germ-free mice may be protected against high fat diet-induced metabolic changes; increased fatty acid metabolism, elevated levels of fasting-induced adipose factor, Fiaf, known as angiopoietin-like protein-4, a secreted lipoprotein lipase inhibitor, and increased AMP-activated protein kinase activity may play a role in this phenomenon [249].

In a randomized, double-blind, parallel, placebo-controlled study to evaluate the effect of prebiotics on plasma levels of gut hormones, 10 healthy subjects received either 16 g prebiotics/day or 16 g dextrin maltose/day for 2 weeks [250]. In subjects following prebiotic treatment, increased gut microbiota fermentation, decreased appetite, improved postprandial glucose responses, and increased plasma levels of GLP-1 and PYY were observed. Everard et al. [251] reported that prebiotic administration led to specific gut microbiota modulation, which improved glucose homeostasis, leptin sensitivity, and target enteroendocrine cell activity in obese and diabetic mice. Furthermore, Muccioli et al. [252] reported that gut microbiota may modulate the intestinal endocannabinoid system tone, which in turn regulates gut permeability and plasma lipopolysaccharide levels.

Taken together, increasing evidence suggests that gut microbiota may be associated with the development of obesity, and that prebiotics which modulate gut microbiota are potential novel treatments for obesity.

## 11. Conclusion

Obesity is a global pandemic and major health burden with associated risk factors of cardiovascular disease and diabetes mellitus. Recent progress has been made in our understanding of energy homeostasis, by characterising an array of complex signalling pathways between the gut and brain. However, no successful pharmacological treatments for obesity have been developed, which can rival the substantial weight loss associated with bariatric surgery. However, bariatric surgery is restricted to patients with morbid obesity, due to its perioperative risks. Modification of the milieu of gut hormones is implicated in the sustained weight loss observed following bypass surgery. In addition, alterations in gut microbial flora following bypass surgery may contribute to weight loss following bariatric surgery [241]. These observations may help to develop a new pharmacological strategy for patients with obesity. The roles of the gut hormones on appetite regulation are summarised in Table 1. Gut hormone-based therapeutics such as GLP-1R agonists and DPP-IV inhibitors have already entered clinical practice, and others are likely to follow. Mimicking the gut hormone milieu observed following bariatric surgery may help us to develop pharmacological therapeutics which lead to substantial and sustained weight loss for patients with obesity.

## Figures and Tables

**Table 1:** The summary of the role of gut hormones on appetite regulation and other actions.

Gut hormones	Feeding	Receptor	Major secretion site	Other actions
PYY_3–36_	↓	Y2	L cells in gut	Delays gastric emptying, inhibits gallbladder contraction, pancreatic exocrine secretions, and gastric acid secretion
PP	↓	Y4, Y5	PP cells in pancreas	Delays gastric emptying, attenuates pancreatic exocrine secretion, and inhibits gallbladder contraction
GLP-1	↓	GLP-1	L cells in gut	Incretin, decreases blood glucose, delays gastric emptying, and neurotrophic effect
OXM	↓	GLP-1	L cells in gut	Inhibits gastric acid secretion and gastric emptying
Glucagon	↓	GCGR	Pancreatic *α* cells	Enhancing physiological response to stress
CCK	↓	CCK 1, 2	I cell of small intestine	Gall bladder contraction, relaxation of sphincter of Oddi, and pancreatic enzyme secretion
Ghrelin	↑	GHS	stomach	Growth hormone secretion, promotes gastric motility, vasodilatation, and increases cardiac contractility
Amylin	↓	AMY1-3	pancreatic *β* cells	Adiposity signals

PYY: peptide YY, PP: pancreatic polypeptide, GLP-1: glucagon-like peptide-1, OXM: oxyntomodulin, CCK: cholecystokinin, GCGR: glucagon receptor.
